# Metabolic Engineering and Synthetic Biology: Synergies, Future, and Challenges

**DOI:** 10.3389/fbioe.2019.00036

**Published:** 2019-03-04

**Authors:** Raúl García-Granados, Jordy Alexis Lerma-Escalera, José R. Morones-Ramírez

**Affiliations:** ^1^Facultad de Ciencias Químicas, Universidad Autónoma de Nuevo León, San Nicolás de los Garza, Mexico; ^2^Centro de Investigación en Biotecnología y Nanotecnología, Facultad de Ciencias Químicas, Universidad Autónoma de Nuevo León, Apodaca, Mexico; ^3^Facultad de Ciencias Biológicas, Universidad Autónoma de Nuevo León, San Nicolás de los Garza, Mexico

**Keywords:** synthetic biology, metabolic engineering, biotechnology, bioengineering, omics analyses

## Abstract

The “-omics” era has brought a new set of tools and methods that have created a significant impact on the development of Metabolic Engineering and Synthetic Biology. These fields, rather than working separately, depend on each other to prosper and achieve their individual goals. Synthetic Biology aims to design libraries of genetic components (promoters, coding sequences, terminators, transcriptional factors and their binding sequences, and more), the assembly of devices, genetic circuits and even organism; in addition to obtaining quantitative information for the creation of models that can predict the behavior of biological systems (Cameron et al., 2014). Metabolic engineering seeks for the optimization of cellular processes, endemic to a specific organism, to produce a compound of interest from a substrate, preferably cheap and simple. It uses different databases, libraries of components and conditions to generate the maximum production rate of a desired chemical compound and avoiding inhibitors and conditions that affect the growth rate and other vital functions in the specific organism to achieve these goals; metabolic fluxes manipulation represents an important alternative (Stephanopoulos, 2012).

While synthetic biology provides the components and information about different biological phenomena, metabolic engineering tries to apply all this information toward the optimization of the biological synthesis trajectory of a desired compound. We can even mention, that some examples of synthetic biology could be also classified as examples of genetic engineering. Despite this, these two areas are dependent on the advances in the actual methods, techniques and tools for DNA modification. The reason is that both areas of research seek to achieve basic requirements such as rational changes in DNA sequences, the generation of specific mutations, the assembly of parts or components in genetic circuits or biosynthetic pathways, the knockout of genes, the integration of DNA pieces in the genome of an organism of interest or in a plasmid (Boyle and Silver, 2012). Although, the PCR and its variants are one of the best tools to generate some of the necessary modifications (it is especially useful for the extraction of fragments of a specific region) it results to be ineffective for other purposes.

The creation of complementary new techniques and methods to achieve other objectives has been vital in the development of both areas, some examples are: Biobricks, Recombinase technologies (integrases), Gibson Assembly, Gap-repair, Lambda-red, MAGE and CRISPR-Cas9, among others (Boyle and Silver, 2012; Stephanopoulos, 2012). During the “-omics” era, the sequencing of both, known and unknown organisms (metagenomics), allows obtaining more and better information from them; the catalog of enzymes and processes was expanded, and certain biological phenomena (for example viruses or phage infections) has been achieved (Goodwin et al., 2016).

Rational design is the introduction of desired mutations into a specific DNA sequence eliding the modification of proteins to improve their catalytic activity, stability or some other property (binding domain specificity); subsequently tools as the Gibson assembly, the biobricks or Golden Gate can assemble the different genetic components to form genetic circuits, expression cassettes or devices (Casini et al., 2015). Finally, the use of Lambda-red, CRISPR-Cas9 and other recombination techniques allow us to insert the different constructions into expression vectors or in strategic loci of the genome of a given organism (Esvelt and Wang, 2013; Liu et al., 2015; Wang et al., 2015).

Some of these technologies can be applied to insert genes of partial or complete metabolic pathways for the synthesis of a specific compound, they can also be used to eliminate genes from the organism that interfere with the synthesis. In addition, these methodologies allow carrying out point mutations that reduce the activity or expression of native proteins to modify the metabolic flux. However, to create all these modifications it is necessary to have certain information about the enzymes that participate in the reactions, the metabolic pathway that includes these reactions and information about the organism where the modifications will be made; because of this, sequencing genomes of organisms, characterization of proteins and metabolic studies provides extremely useful tools and information (Figure 1).

**Figure 1:**
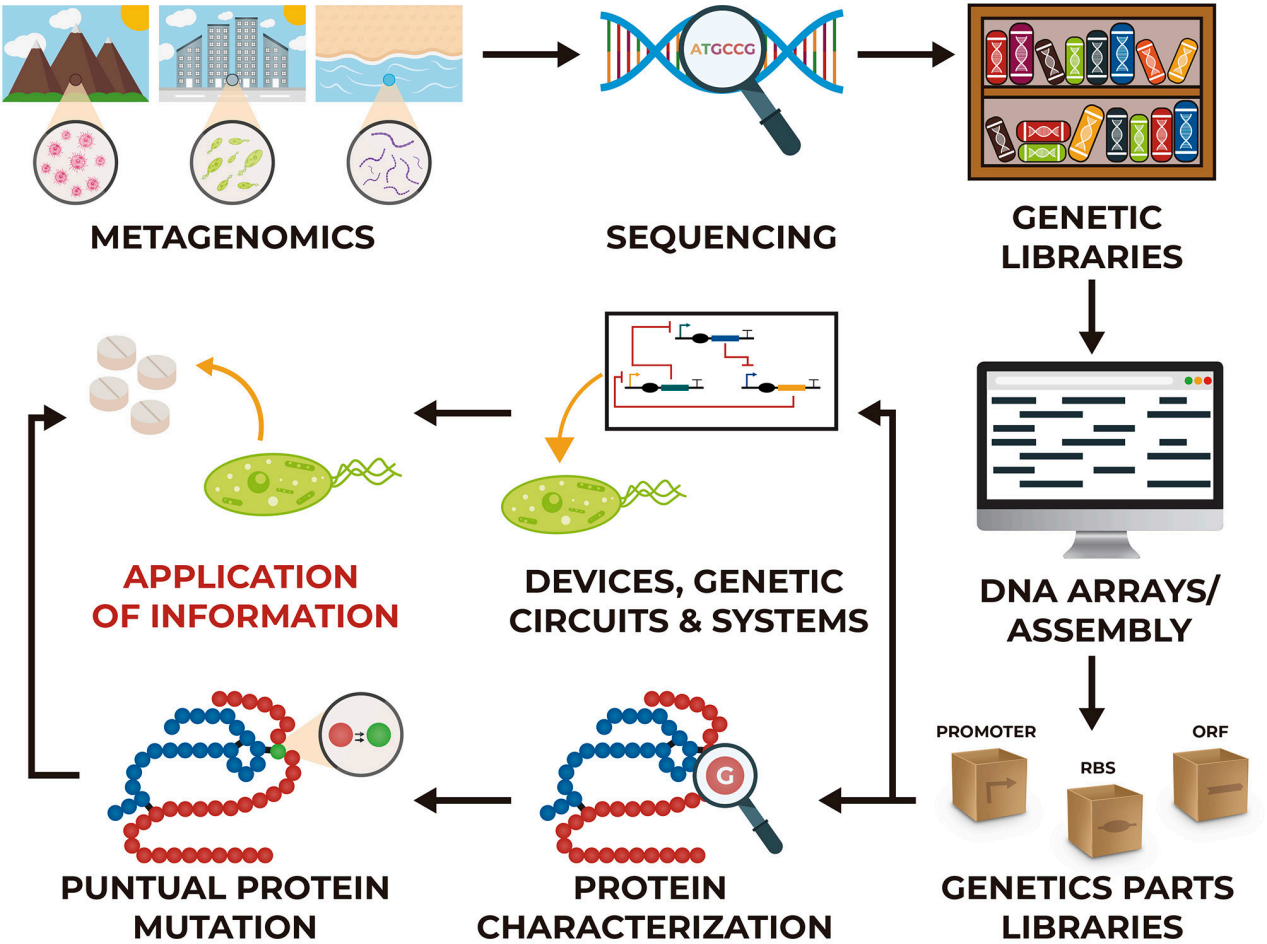
Different stages for the development of synthetic biology and metabolic engineering projects.

Therefore, one of the main issues is the lack of information; very few organisms have been sequenced and characterized at different levels (genome, transcriptome and metabolome); making the genetic modifications a difficult task to achieve. Added to this, the techniques and methods that currently exist are not effective or have low performance rates that depend on the organism. The creation of plasmids capable of being used in different organisms (shuttle vectors) will aid in the solution of this problem but sequencing remains a necessary step. With this information we could discover new proteins or metabolic pathways that could be useful for higher compound production titles (enzymes with better production rates or simpler metabolic pathways); we could even describe new biological phenomena that are useful for the modification of DNA sequences.

Another important point related to the information available, is the selection of the chassis (or microorganism on which we will working on). The basic information and techniques available, as well as the special qualities (specific metabolic pathways or certain resistant to conditions) are important criteria when we are choosing the chassis and could facilitate the development of a project (Brophy and Voigt, 2014; Khoury et al., 2014).

A problem related to the choice of the chassis and the modification of both the metabolic pathway and metabolic flux, is the production of toxic intermediates or the accumulation of an intermediate that could cause the inhibition of the route (feedback). The production of certain compounds can result in toxicity for the microorganism that produce it, especially when is handled at large scales (Sopko et al., 2006; Förster and Gescher, 2014). In these cases, a correct design of the bioreactor is usually helpful, removing the toxic compound before it reaches a threshold concentration of damage, and therefore improving significantly the production titles. Here appears another challenge for both areas, to discover a microorganism that can resist a greater concentration of that compound, or to discover and adapt the mechanism of resistance from organism to another (Keasling, 2012). However, to avoid the accumulation of an intermediate in any of the steps from the metabolic pathway, it is necessary to determine the rates of synthesis and production of each of the compounds; once you have this information, tinkering with the strength of promoters, RBS, transcriptional factors, or in some cases, the growth conditions allow us to avoid this problem, but the main problem still relies in having these information available.

The most important challenge for both areas is the obtention of quantitative data from different biological phenomena (Le Novère, 2015). We need more projects that are responsible for characterizing biological components; like the strength of promoters, RBSs, terminators; as well as the behavior of certain phenomena (transcription and translation levels of genes, average life of transcripts and proteins, catalytic activity, repressive force). Also, the creation of genetic circuits and devices has helped with the description of more complex behaviors and the understanding of previously mentioned phenomena (Canton et al., 2008).

All these data allow the creation of mathematical and computational models that provides us with a better understanding of the behavior of the system; and helps to have a visualization of expected behaviors, approximate productions, selection of conditions, etc. (Hwang et al., 2009). There is a diversity of works focused on biological simulation: Karr et al. (2012) report a whole-cell computational model of *Mycoplasma genitalium*, including the molecular components and their interactions by combining different mathematical and computational approaches (ODEs with Boolean, probabilistic and constraint-based submodels). Moreover, O'Brien et al. (2015) mentions the different applications that the mathematical models and the computational simulation offers: simulating gene and reaction knockouts, comparing inputs and outputs, iterative improvement, gap-filling approaches, discovery of regulatory interactions, quantification of predictable phenotypes from optimality principles, model-based design of environmental and genetic perturbations, to mention some.

The application of the mathematical models could be a powerful tool to understand biological systems and the computational simulations could give us a better perspective of the processes and the changes that occur or of the predictions. Technologies such as the Machine Learning, the Big Data and the Artificial Intelligence are being of great help for the analysis of biological data. Different methods (Artificial Neural Nets, Bayesian, Decision Tree and Random Forrest, Multi-layer perceptron (MLP), Radial basis function (RBF) networks, Support Vector Machines, K-means, Farthest First, Density Base Clustering, etc.) are being used for microarrays, study of diseases, epidemiology; generating useful information for different researches (Pirooznia et al., 2008; Libbrecht and Noble, 2015). The use of quantum computers represents a very important tool for the development of these simulations and methods; these computers process the information in qubits (can represent either 1 or 0 or combinations of them, known as superposition) so it has a greater processing scope, something very valuable when you work with large amounts of information; the first big breakthrough has been the IBM to take public sale the first commercial quantum computer in history (the IBM Q System One). It is only a matter of time to discover the power that gives different researchers the analysis of information that provides the quantum computer in the biological and health area.

Both metabolic engineering and synthetic biology are two promising areas that have made great advances in biotechnology and have contributed significantly toward the resolution of problems in production of drugs, vaccines, chemical compounds, etc. (Khalil and Collins, 2010). In addition, these fields have advanced our knowledge regarding life function. Despite all these advances, it is still necessary to continue collecting information regarding functioning of cells and living organisms and discover new species of microorganisms that could aid in the development of new techniques and methods. Finally, synthesizing long sequences is often problematic because of the margin of error that exists with current chemical synthesis techniques (approximately 1 error in every 1,000 base pairs); the search for new nucleotide synthesis techniques (such as TdT-dNTP or enzymatic synthesis) or the improvement of the current chemical synthesis of nucleotides are aspects to be improved and could be very useful for both areas, especially because it opens the opportunity for the synthesis of whole genomes, vectors or artificial chromosomes (Kosuri and Church, 2014; Hughes and Ellington, 2017).

With all these tools at our disposal, we will be able to optimize microorganisms as small factories that allow us to obtain higher rates of yield and production of a chemical compound, preferably using simple substrates.

## Author Contributions

RG-G, JL-E, and JM-R all contributed toward the writing and editing of this manuscript.

### Conflict of Interest Statement

The authors declare that the research was conducted in the absence of any commercial or financial relationships that could be construed as a potential conflict of interest.
